# Redefining thymus medulla specialization for central tolerance

**DOI:** 10.1084/jem.20171000

**Published:** 2017-11-06

**Authors:** Emilie J. Cosway, Beth Lucas, Kieran D. James, Sonia M. Parnell, Manuela Carvalho-Gaspar, Andrea J. White, Alexei V. Tumanov, William E. Jenkinson, Graham Anderson

**Affiliations:** 1 Institute for Immunology and Immunotherapy, College of Medical and Dental Sciences, Medical School, University of Birmingham, Birmingham, England, UK; 2 Department of Microbiology, Immunology, and Molecular Genetics, University of Texas Health Science Center at San Antonio, San Antonio, TX

## Abstract

During αβT cell development, the thymus medulla represents an essential microenvironment for T cell tolerance. This functional specialization is attributed to its typical organized topology consisting of a branching structure that contains medullary thymic epithelial cell (mTEC) networks to support negative selection and Foxp3^+^ T-regulatory cell (T-reg) development. Here, by performing TEC-specific deletion of the thymus medulla regulator lymphotoxin β receptor (LTβR), we show that thymic tolerance mechanisms operate independently of LTβR-mediated mTEC development and organization. Consistent with this, mTECs continue to express Fezf2 and Aire, regulators of intrathymic self-antigens, and support T-reg development despite loss of LTβR-mediated medulla organogenesis. Moreover, we demonstrate that LTβR controls thymic tolerance by regulating the frequency and makeup of intrathymic dendritic cells (DCs) required for effective thymocyte negative selection. In all, our study demonstrates that thymus medulla specialization for thymic tolerance segregates from medulla organogenesis and instead involves LTβR-mediated regulation of the thymic DC pool.

## Abstract (precis)

The thymus medulla prevents T cell–driven autoimmunity via central tolerance. Cosway et al. show that this specialization occurs independently of the topology that classically defines its structure and demonstrate that medulla function requires LTβR-mediated regulation of dendritic cells for negative selection.

## Introduction

The thymus generates αβT cells that respond to foreign antigens presented by self-MHC molecules (Boehm, 2008). During intrathymic development, thymocytes express a randomly generated αβTCR repertoire that is screened to bias thymus function toward self-tolerant T cell production (Kappler et al., 1987; Jenkinson et al., 1989; Kishimoto and Sprent, 1997). This requires thymic selection mechanisms involving stromal cells in anatomically distinct areas (Takahama et al., 2008). In the cortex, cortical thymic epithelial cells (TECs [cTECs]) trigger positive selection (Anderson et al., 1994; Laufer et al., 1996; Murata et al., 2007). This process also induces expression of CCR4 and CCR7 (Ueno et al., 2004; Ehrlich et al., 2009; Cowan et al., 2014; Hu et al., 2015) to allow newly selected thymocytes access to the medulla. Here, negative selection eliminates thymocytes bearing high-affinity αβTCRs via apoptosis (Daniels et al., 2006; Klein et al., 2014). The medulla also supports Foxp3^+^ T-regulatory (T-reg) development (Aschenbrenner et al., 2007; Perry et al., 2014; Malhotra et al., 2016), and although mechanisms discriminating these processes are unclear, both medullary TECs (mTECs) and DCs are important (Cowan et al., 2013; Perry et al., 2014; Herbin et al., 2016).

Several features of the medulla may explain its specialization for tolerance. First, formation from clonally derived islets creates a complex 3D topology which, in WT mice, consists of small areas that may be connected to a larger medullary compartment. This process is initiated during organogenesis, is maintained in adulthood (Rodewald et al., 2001; Boehm et al., 2003; Irla et al., 2013), and provides lymphostromal interactions for single-positive thymocytes (Anderson and Takahama, 2012). Second, the medulla houses DCs, with Aire^+^ mTECs producing XCL1 to control DC positioning for T-reg generation (Lei et al., 2011). Finally, specialized mTEC subsets express key genes that collectively regulate tolerance. Of these, Aire and Fezf2 are the two known regulators of intrathymic expression of tissue-restricted antigens (TRAs). Absence of either Aire (Anderson et al., 2002) or Fezf2 (Takaba et al., 2015) results in tolerance breakdown, which fits well with their ability to regulate differing TRAs. Although Aire is controlled by RANK (Rossi et al., 2007; Akiyama et al., 2008; Hikosaka et al., 2008), lymphotoxin β receptor (LTβR) was reported as an essential regulator of Fezf2 expression in mTECs (Takaba et al., 2015). Indeed, both *Rank^−/−^* (Rossi et al., 2007; Akiyama et al., 2008; Hikosaka et al., 2008) and *Ltbr*^−/−^ (Boehm et al., 2003; Venanzi et al., 2007; Zhu et al., 2007; White et al., 2010) mice demonstrate defective medulla formation and loss of tolerance. Collectively, these findings suggest a dual requirement for RANK/Aire and LTβR/Fezf2 pathways during T cell tolerance. Furthermore, they help form current models in which mTEC organization and development are prerequisites of tolerance induction, with medulla abnormalities being causative factors in tolerance breakdown (Akiyama et al., 2015; Abramson and Anderson, 2017).

Here, we have explored mechanisms that control the thymus medulla and determine its ability to mediate tolerance. Specifically, we examined the relationship between LTβR and coordination of mTECs and DCs for negative selection and T-reg generation. We show that despite a profound perturbation of mTECs caused by TEC-specific deletion of LTβR, T cell tolerance remains intact, challenging the notion that thymic tolerance is determined by medulla organization and development. Rather, we show that an essential feature of medulla function involves LTβR-mediated control of the thymic DC pool for negative selection. In all, our study separates the process of medulla formation from its control of thymic tolerance and identifies a new role for LTβR in the regulation of thymus function.

## Results and discussion

### TEC-restricted deletion of LTβR dissociates medulla topology from tolerance induction

**Figure 1.**
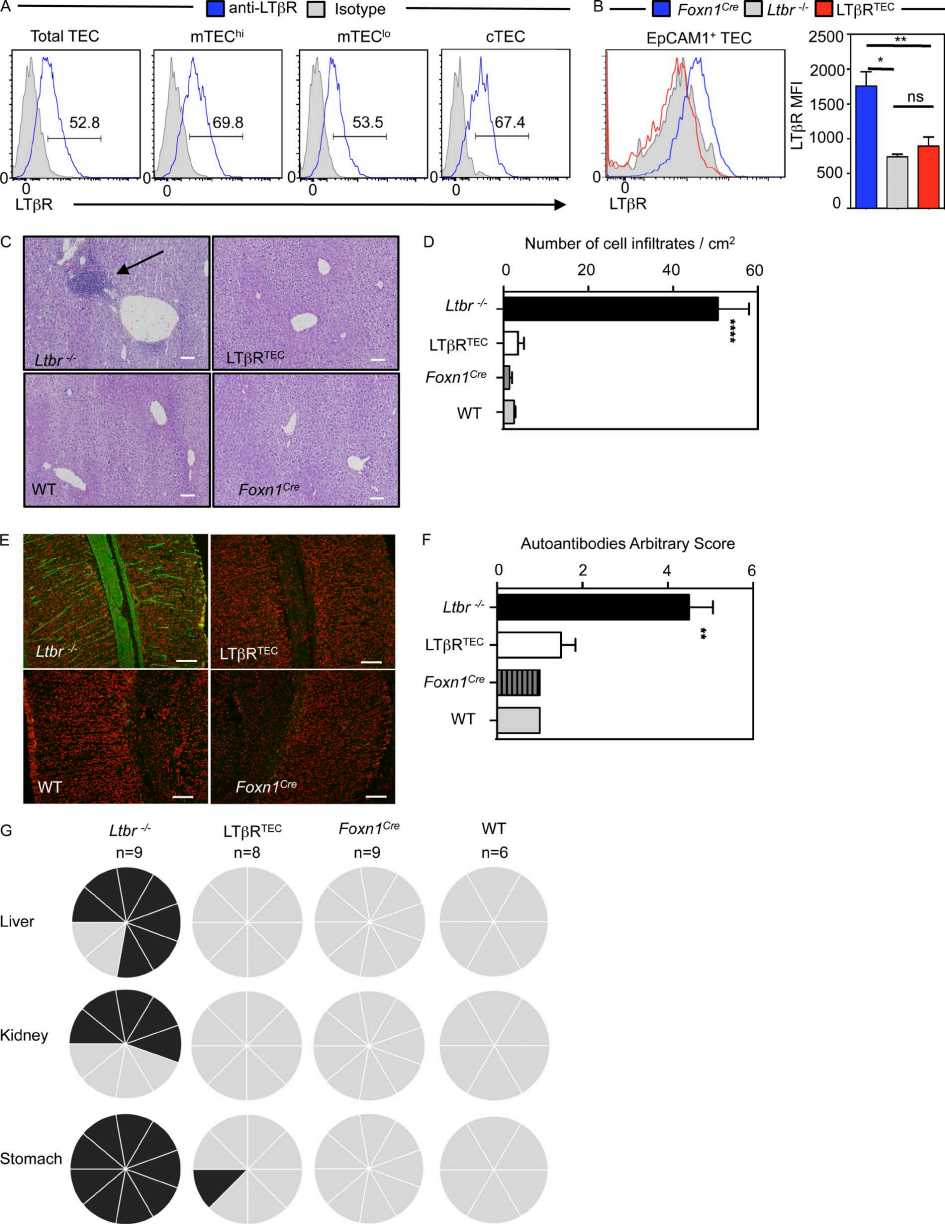
**LTβR deletion in thymic epithelium corrects the autoimmunity in germline *Ltbr^−/−^* mice.** (A) LTβR in WT EpCAM1^+^ TECs, Ly51^+^ cTECs, Ly51^−^MHCII^lo^CD80^lo^ mTEC^lo^ cells, and Ly51^−^MHCII^hi^CD80^hi^ mTEC^hi^ cells; gray histogram is isotype control staining. (B) Anti-LTβR staining in TECs from indicated strains. Data represent two experiments, *n* ≥ 4 mice. (C) Liver sections from mice at 8–12 wk of age. Arrow indicates lymphocytic infiltrates. Bars, 100 µm. (D) Quantitation of infiltrates in B. Data from ≥3 mice from two experiments. (E) WT sections incubated with 1/80 sera to detect autoantibodies (green); DAPI in red; staining on stomach shown as an example. Bars, 100 µm. (F) Quantification of autoantibody staining in stomach. Data represent at least two experiments, *n* ≥ 5 mice. (G) Summary of autoantibody detection in various tissues. Each segment represents one mouse; black denotes positive staining. Error bars indicate SEM. *, P < 0.05; **, P < 0.01; ****, P < 0.0001.

Normal programs of mTEC development and medulla formation are seen to be essential for the specialized function of this site. The TNF receptor superfamily (TNFRSF) member LTβR is a key regulator of thymic microenvironments and intrathymic tolerance, and its expression is readily detectable in multiple TEC subsets (Fig. 1 A). However, in studies using germline *Ltbr^−/−^* models, it is unclear whether effects on tolerance are directly attributable to alterations in TEC development or function. To examine this, we crossed *Foxn1^Cre^* mice (Gordon et al., 2007) with mice carrying floxed alleles of LTβR (Wang et al., 2010) to create LTβR^TEC^ mice. Importantly, LTβR expression by EpCAM1^+^ TEC was absent in *Ltbr^−^*^/^*^−^* mice and LTβR^TEC^ mice (Fig. 1 B), demonstrating the effectiveness of this model to examine the relationship between medulla function and tolerance.

**Figure 2.**
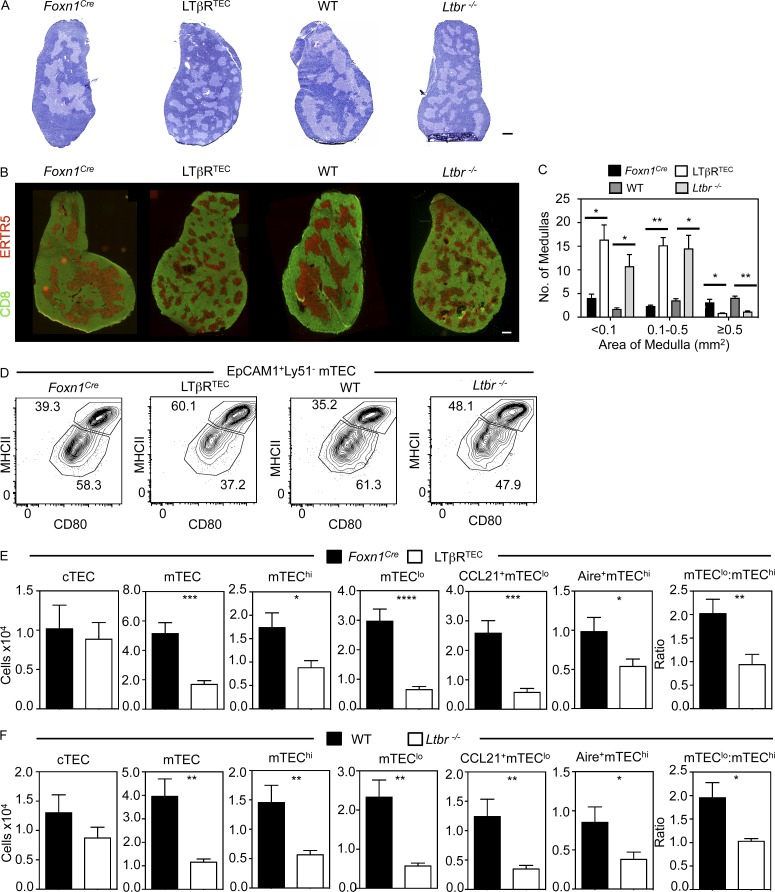
**Defective mTEC development and medulla topology in LTβR^TEC^ mice.** (A) Thymic architecture in indicated mouse strains. Bar, 500 µm. (B) Sections stained with anti-CD8 (green) to detect cortex and ERTR5 (red) to detect medulla. Bar, 500 µm. Images represent ≥4 mice. (C) Quantitation of medulla areas in sections. Data are means of three sections per mouse, *n* = 3 per strain. Data from three separate experiments. (D) CD80/MHCII in EpCAM1^+^Ly51^−^ mTECs. (E and F) TEC quantitation in *Foxn1^Cre^*, LTβR^TEC^, WT, and *Ltbr^−/−^* mice. Data from four experiments, *n* = 12. Error bars indicate SEM. *, P < 0.05; **, P < 0.01; ***, P < 0.001; ****, P < 0.0001.

A key feature of *Ltbr^−/−^* mice is a breakdown in central tolerance. This manifests as lymphocytic infiltrates in multiple organs and the presence of serum autoantibodies (Boehm et al., 2003; Venanzi et al., 2007; Zhu et al., 2007; Martins et al., 2008). Because disruption of thymic tolerance in *Ltbr^−/−^* mice correlates with defective medulla formation and mTEC development (Boehm et al., 2003), we examined thymic tolerance in LTβR^TEC^ mice. Although several tissues (liver, kidney, stomach, salivary gland) from *Ltbr^−/−^* mice showed signs of autoimmunity including cell infiltrates, positive autoantibody staining, and presence of activated T cells, these features were markedly absent from LTβR^TEC^ mice (Fig. 1, C–G; and Fig. S1). Notably, the lack of measurable autoimmunity in LTβR^TEC^ mice occurred despite the presence of intact secondary lymphoid tissues that are defective in *Ltbr^−/−^* mice (not depicted). We examined LTβR^TEC^ mice up to 5 mo of age and still failed to observe signs of autoimmunity (not depicted). Importantly, compared with medulla organization in WT and *Foxn1^Cre^* controls, both *Ltbr^−/−^* and LTβR^TEC^ mice showed disruption of the typical 3D medulla architecture (Fig. 2, A and B). Indeed, large ERTR5^+^ mTEC areas present in control mice were absent, and ERTR5^+^ areas were smaller and scattered throughout thymic sections (Fig. 2 B). Thus, *Ltbr^−/−^* and LTβR^TEC^ mice both showed a significant decrease in the number of large (≥0.5-mm^2^) medulla areas per thymus section and an increased number of smaller (<0.1-mm^2^ and 0.1–0.5-mm^2^) medullas (Fig. 2 C). Interestingly, despite detectable LTβR expression by cTECs (Fig. 1 A), we saw no significant alterations in cTEC numbers in *Ltbr^−/−^* and LTβR^TEC^ mice (Fig. 2, E and F). In contrast, both mice had defects in mTECs, including reduced numbers of mTEC^low^, mTEC^hi^, and subsets of CCL21^+^ and Aire^+^ cells (Fig. 2, D–F). Thus, in LTβR^TEC^ mice where TEC specific deletion of LTβR recapitulates the medullary disorganization in *Ltbr^−/−^* mice, T cell tolerance is maintained. Collectively, these findings indicate that autoimmunity is not a direct consequence of medulla dysgenesis and is distinct from the impact of LTβR on mTECs.

### RANK controls both known intrathymic regulators of promiscuous gene expression

**Figure 3.**
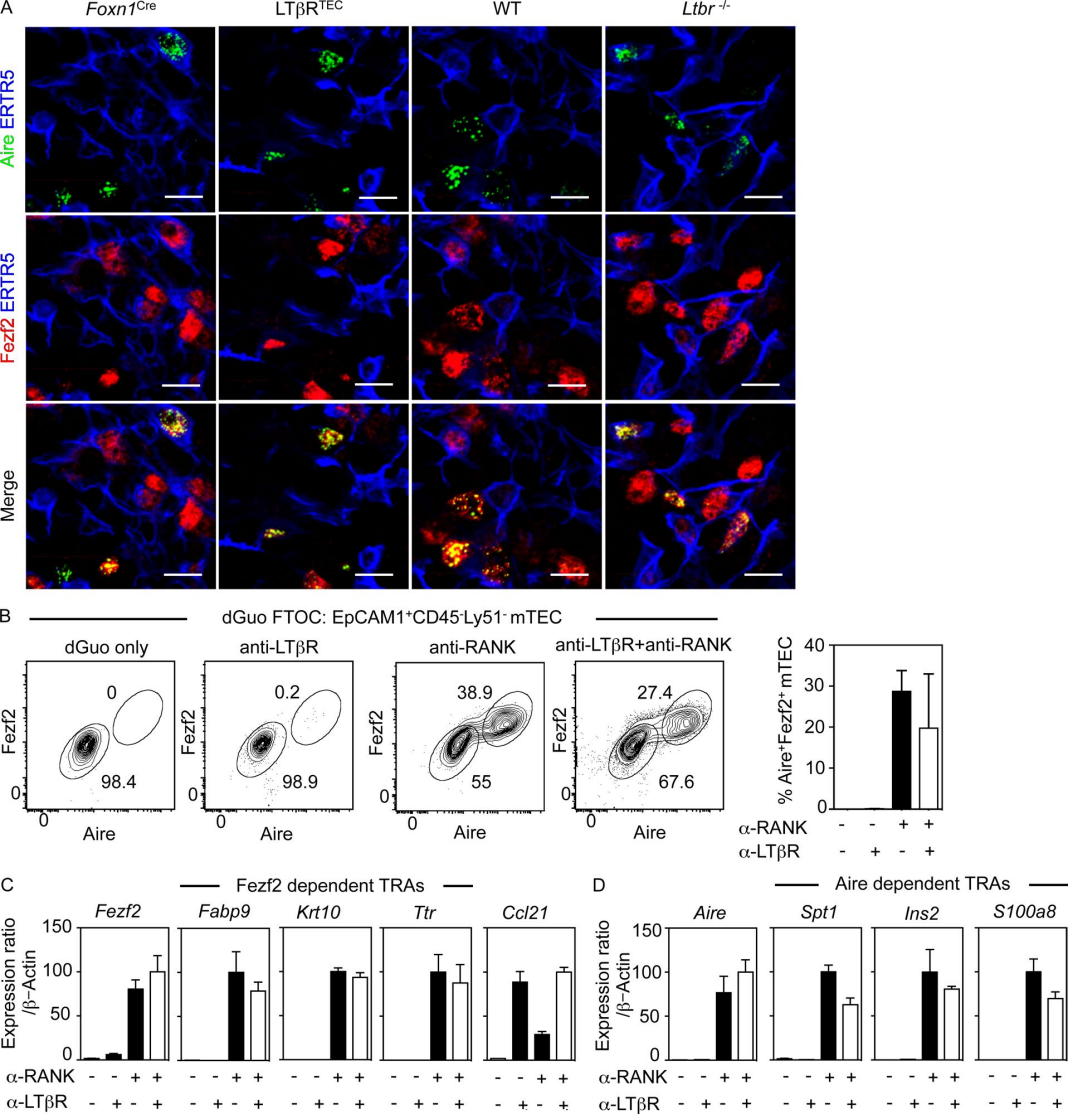
**RANK controls intrathymic regulators of promiscuous gene expression.** (A) Confocal images of thymus sections stained for Aire (green), ERTR5 (blue), and Fezf2 (red). Bars, 10 µm. Data represent three experiments, *n* ≥ 6. (B) Aire and Fezf2 in Ly51^−^ mTECs in dGuo FTOCs treated as indicated. Graph shows percentage Aire^+^Fezf2^+^ mTECs. Data from at least three separate experiments. (C and D) qPCR of indicated genes in anti-RANK/anti-LTβR stimulated dGuo FTOCs. Data from at least two independent experiments. Error bars represent SEM.

Significant to our findings is a study suggesting that LTβR controls mTEC expression of Fezf2, a transcription factor that regulates intrathymic TRAs (Takaba et al., 2015). Indeed, absence of Fezf2^+^ mTEC from *Ltbr^−/−^* mice was reported as a major factor in loss of tolerance in these mice. Importantly, using the same anti-Fezf2 antibody (Takaba et al., 2015), we detected Fezf2^+^ mTEC in both *Ltbr^−/−^* and LTβR^TEC^ mice (Fig. 3 A). Moreover, anti-LTβR stimulation of dGuo fetal thymus organ culture (FTOC) did not induce expression of Fezf2 (Fig. 3, B and C) nor the previously reported Fezf2-dependent TRAs *Fabp9*, *Krt10*, and *Ttr* (Fig. 3 C; Takaba et al., 2015). Importantly, this failure was not caused by ineffective stimulation, because anti-LTβR induced expression of *Ccl21* mRNA (Fig. 3 C). In contrast, anti-RANK stimulation induced high levels of both Fezf2 and Aire in mTECs (Fig. 3, B–D), as well as Aire-dependent and Fezf2-dependent TRAs (Fig. 3, C and D). Expression of Aire, Fezf2, and associated TRAs was not augmented by combined RANK and LTβR stimulation (Fig. 3, B–D). Consistent with the expression pattern of Aire (Gray et al., 2007), Fezf2 was detectable only in mTEC^hi^ cells (not depicted). Thus, although LTβR influences mTEC development and organization (Boehm et al., 2003; Lkhagvasuren et al., 2013; Wu et al., 2017), it is not required for generation of Fezf2^+^ mTECs. Rather, RANK represents a key regulator of both Aire^+^ and Fezf2^+^ mTECs. This demonstrates that the requirement for RANK in thymic tolerance is linked to control of mTEC development, including Aire and now Fezf2 expression, and emphasizes that the role of LTβR in central tolerance is distinct from its ability to control intrathymic TRA expression. This is reinforced by our finding that LTβR^TEC^ mice do not show symptoms of autoimmunity and suggests that despite a reduction in the number of TRA-producing cells, the capacity for mTEC-dependent self-antigen production in LTβR^TEC^ mice exceeds any threshold requirement for tolerance induction in the naturally diverse αβTCR repertoire.

### Foxp3^+^ T-reg production occurs independently of LTβR and medulla organization

**Figure 4.**
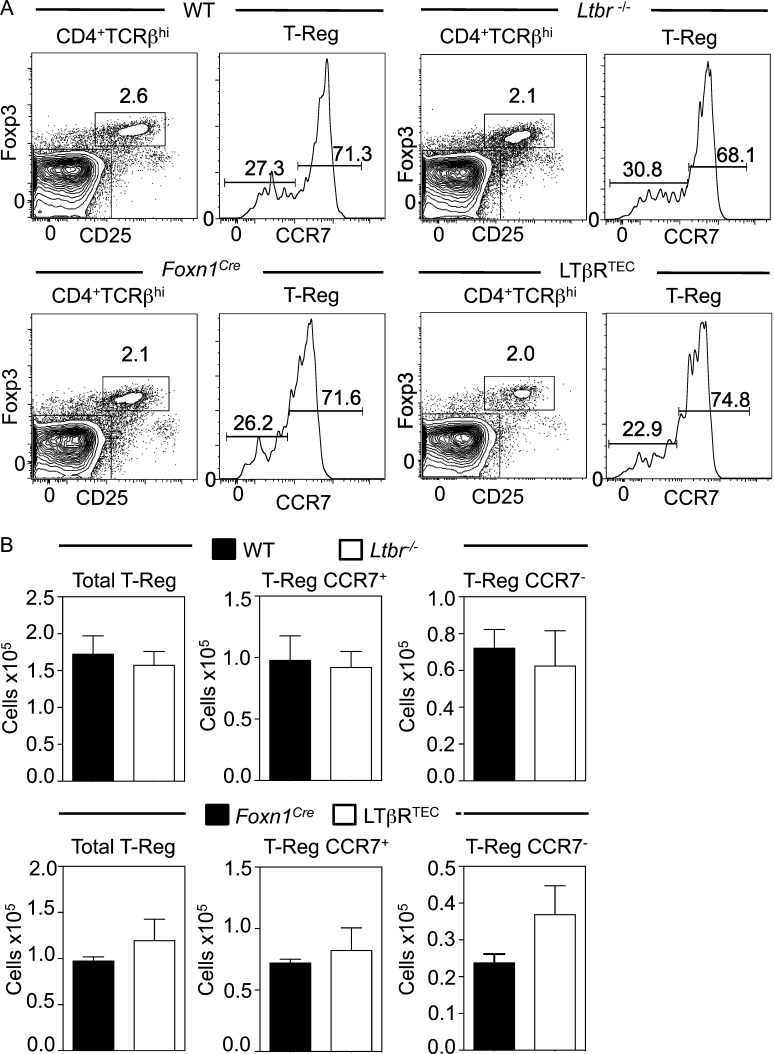
**De novo Foxp3^+^ T-reg development occurs independently of LTβR.** (A) Analysis of CD25^+^Foxp3^+^CD4^+^TCRβ^+^ thymocytes, with CCR7 expression to identify newly selected CCR7^+^ Foxp3^+^ T-reg. (B) Absolute numbers of CCR7^+^ and CCR7^−^ Foxp3^+^ T-reg. Data typical of at least seven mice from three separate experiments. Error bars represent SEM.

In the medulla, interactions between thymocytes and mTECs/DCs result in Foxp3^+^ T-reg development (Tai et al., 2013; Perry et al., 2014). Given the essential requirement for mTECs in Foxp3^+^ T-reg development (Cowan et al., 2013), we analyzed this process in *Ltbr*^−/−^ and LTβR^TEC^ mice. Importantly, and in contrast to previous studies (Zhu et al., 2007; Martins et al., 2008), we separated total thymic T-reg using CCR7 to discriminate de novo from recirculating T-reg (Cowan et al., 2016). In *Ltbr*^−/−^ and LTβR^TEC^ mice, both proportions and absolute numbers of newly generated CCR7^+^Foxp3^+^ T-reg were comparable to those of control mice (Fig. 4, A and B). Thus, Foxp3^+^ T cell development is not dependent on LTβR-mediated control of the medulla, suggesting that failure of tolerance in *Ltbr*^−/−^ mice is not caused by defective Foxp3^+^ T-reg generation secondary to disruption of medulla structure.

### LTβR controls the thymic DC pool for negative selection

**Figure 5.**
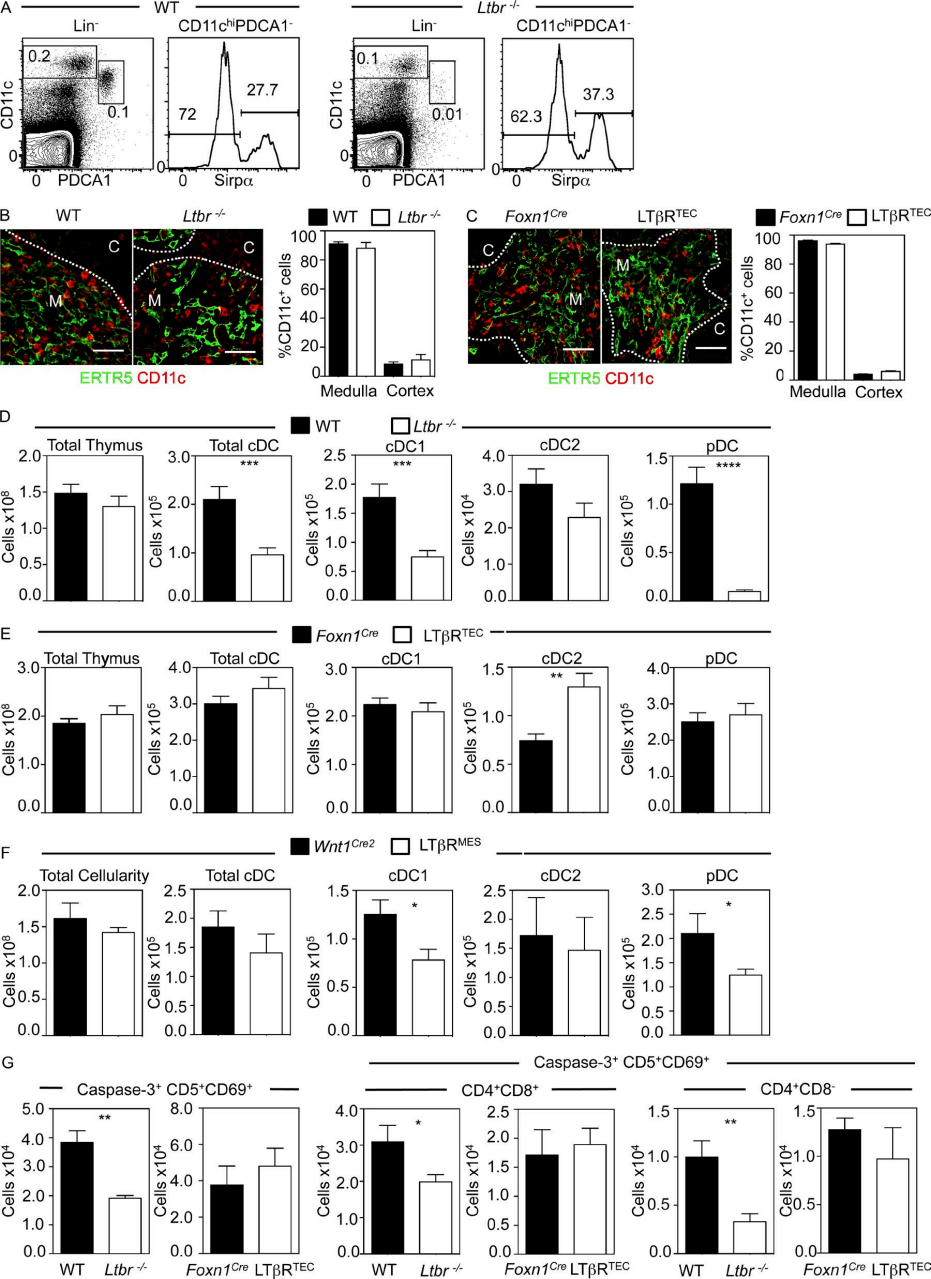
**LTβR controls formation of the thymic DC pool for negative selection.** (A) cDC1, cDC2, and pDCs in Lin^−^ thymus preparations. (B and C) CD11c^+^ DCs in ERTR5^+^ areas of indicated mice. Bars, 50 µm. Data from three experiments, *n* ≥ 6. (D and E) Thymic DCs in WT/*Ltbr^−/−^* and LTβR^TEC^/*Foxn1^Cre^* mice; *n* = 11 from three experiments. (F) Thymic DC numbers in *Wnt1^Cre2^* and LTβR^MES^ mice; data from three experiments, *n* ≥ 6. (G) Numbers of total, DP, and CD4^+^ CD5^+^CD69^+^Caspase3^+^ thymocytes. Data obtained from at least two experiments where *n* ≥ 5 for all strains. Error bars indicate SEM. *, P < 0.05; **, P < 0.01; ***, P < 0.001; ****, P < 0.0001.

Given the importance of multiple DC subsets in thymic tolerance (Proietto et al., 2008; Hadeiba et al., 2012; Perry et al., 2014), we examined thymic DCs in *Ltbr^−/−^* and LTβR^TEC^ mice. We used flow cytometric analysis of digested thymuses to identify PDCA1^+^CD11c^low^ plasmacytoid DCs (pDCs) and PDCA1^−^CD11c^+^ conventional DC (cDC) subsets in the CD3^−^CD19^−^NK1.1^−^ (Lin^−^) fraction. CD11c^+^ cDCs were further subdivided into SIRPα^−^ cDC1 and SIRPα^+^ cDC2 cells (Fig. 5 A). Although DCs in both WT and *Ltbr^−/−^* mice were predominantly located in the medulla (Fig. 5 B), we saw alterations in the thymic DC pool size of *Ltbr^−/−^* mice, with numbers of both pDCs and cDCs significantly reduced compared with WT (Fig. 5 D). This impact on cDCs mapped to a selective reduction in cDC1 cells, with comparable cDC2 numbers in WT and *Ltbr^−/−^* mice (Fig. 5 D). Importantly, we saw no differences in BrdU incorporation in thymic DCs from *Ltbr*^−/−^ and LTβR^TEC^ mice (Fig. S2), suggesting that the diminished numbers were not caused by reduced DC proliferation. Thus, in *Ltbr^−/−^* mice where both mTECs and tolerance are defective, LTβR controls the size and makeup of the thymic DC pool. When we analyzed thymic DCs in LTβR^TEC^ mice, where mTEC development is impaired but tolerance is maintained, DCs were located throughout ERTR5^+^ medullary areas in a manner comparable to control mice (Fig. 5 C). However, and in contrast to *Ltbr^−/−^* mice, we found no reduction in thymic DCs in LTβR^TEC^ mice (Fig. 5 E). Indeed, we saw increased cDC2 cells in LTβR^TEC^ mice compared with *Foxn1^Cre^* controls. Although the reasons for this are currently unclear, one possibility is that LTβR expression by TECs may act to suppress intrathymic cDC2 numbers as part of its role in controlling the size and makeup of the intrathymic DC pool. Collectively, analysis of intrathymic DCs in *Ltbr^−/−^* and LTβR^TEC^ mice shows that the requirement for LTβR in thymic tolerance correlates with a reduction in thymic DC frequency that is unconnected to LTβR-mediated mTEC regulation.

LTβR controls splenic DCs in a cell-autonomous manner (Kabashima et al., 2005; Wang et al., 2005). We generated BM chimeras using WT and *Ltbr*^−/−^ host/donor combinations, and this confirmed the cell-intrinsic requirement for LTβR by splenic DCs (Fig. S3). Importantly, however, thymic DCs were significantly reduced in WT:*Ltbr^−/−^* but not *Ltbr*^−/−^:WT chimeras (Fig. S3). Thus, the requirement for LTβR by thymic DCs is non–cell autonomous and instead maps to radioresistant stroma, with no reduction in thymic DC numbers in LTβR^TEC^ mice, indicating a role for LTβR expression by non-TEC stroma. As the thymic mesenchyme has been implicated in various aspects of thymus function (Jenkinson et al., 2003, 2007), we investigated whether these cells play a role in controlling thymic DCs. Thus, we crossed *Wnt1^Cre2^* mice, in which Cre is expressed by neural crest derived mesenchymal cells (Lewis et al., 2013), with *Ltbr^fl/fl^* mice to generate LTβR^MES^ mice in which thymic deletion of LTβR is limited to the mesenchyme. Interestingly, we saw a significant and selective decrease in both cDC1 cells and pDCs in LTβR^MES^ mice compared with *Wnt1^Cre2^* controls (Fig. 5 F), a pattern that mirrors the thymic DC defect in *Ltbr^−/−^* mice. Collectively, comparison of the cellular regulators of thymic tolerance in *Ltbr^−/−^* and LTβR^TEC^ mice and analysis of their autoimmune status indicates that LTβR controls formation of the thymic DC pool via a mechanism distinct from its regulation of mTEC development and medulla formation. Because a key role of DCs is clonal deletion of autoreactive thymocytes (Gallegos and Bevan, 2004; Bonasio et al., 2006), we analyzed the frequency of Caspase-3^+^CD5^+^CD69^+^ thymocytes, representing cells undergoing negative selection in the naturally diverse WT αβTCR repertoire (Stritesky et al., 2013). Compared with WT, *Ltbr^−/−^* mice contained fewer Caspase-3^+^CD5^+^CD69^+^ thymocytes, indicating a reduction in negative selection (Fig. 5 G). Moreover, combined analysis of CD4, CD8, and Caspase-3 expression showed a greater reduction in the number of CD4^+^ thymocytes undergoing negative selection compared with double-positive (DP) thymocytes (Fig. 5 G). In contrast, in LTβR^TEC^ mice, in which self-tolerance and thymic DCs are maintained, the frequency of total, DP, or CD4^+^ Caspase-3^+^CD5^+^CD69^+^ thymocytes was not changed (Fig. 5 G). Thus, breakdown of thymic tolerance in *Ltbr^−/−^* but not LTβR^TEC^ mice correlates with reductions in both thymic DCs and the frequency of thymocytes undergoing negative selection.

The thymus medulla is a specialized microenvironment essential for T cell tolerance. This has been attributed to its 3D organization and the presence of multiple mTEC subsets residing within a complex structure consisting of multiple islets that may branch from a larger medullary core (Irla et al., 2013). This anatomical specialization is thought to foster mTEC and DC function and limit autoimmune responses via negative selection and Foxp3^+^ T-reg development. Here, we show that TEC-specific deletion of LTβR disrupts medulla formation and mTEC development and limits mTEC availability. Although it is currently unclear whether LTβR expression by cTECs plays a functional role in T cell development, it is important to note that absence of LTβR does not alter cTEC numbers, suggesting that it may not play an essential role in cTEC development. Importantly, and despite the alterations in mTECs caused by TEC-specific loss of LTβR, thymus dysgenesis does not alter its ability to impose T cell tolerance mechanisms. Thus, our data suggest that “form ever follows function” models (Sullivan, 1896) do not necessarily apply to the thymus medulla. Although mTECs are an essential requirement for tolerance induction (Cowan et al., 2013), we show that quantitative limitation of their availability, and a loss of typical thymus architecture, still allows the medulla to operate as a tolerizing site. This finding is important in understanding how the medulla imposes tolerance mechanisms. For example, although TCR transgenic T-reg development is limited by intrathymic niche availability (Bautista et al., 2009; DiPaolo and Shevach, 2009; Leung et al., 2009), mTEC loss in LTβR^TEC^ mice does not impair Foxp3^+^ T-reg development or negative selection. Thus, for the naturally diverse αβTCR repertoire, intrathymic niche availability does not rate-limit the ability of the medulla to support both dominant and recessive tolerance. Furthermore, despite reduced mTECs in LTβR^TEC^ mice, frequencies of mature CD4^+^ and CD8^+^ thymocytes are unaltered (unpublished data). Whether this reflects changes in thymocyte motility/dwell time that compensate for reduced mTEC availability is not known. In addition, because LTβR influences mTEC shape (Boehm et al., 2003), alterations in mTEC cell surface area in LTβR^TEC^ mice may alter thymocyte interactions. Interestingly, however, the segmented nature of the medulla in LTβR^TEC^ mice does not lead to tolerance breakdown, which is perhaps consistent with a similar distribution of medullary islands in juvenile mice (Rodewald et al., 2001) and the confinement of thymocytes to tolerance-inducing medulla subunits (Le Borgne et al., 2009).

Perhaps most significant to current understanding of mTEC development is our finding that LTβR does not control expression of Fezf2, a regulator of intrathymic TRAs. This contrasts with a recent study (Takaba et al., 2015), and although the reasons for this difference are not clear, it is important to note that our finding remains compatible with the idea that Fezf2 regulates tolerance via control of TRA expression. Importantly, we find that Fezf2 expression in mTECs is regulated by RANK-mediated signaling events. Thus, RANK signaling controls expression of both Aire and Fezf2, the two known regulators of intrathymic TRAs, which demonstrates the requirement for this TNFRSF member in thymic tolerance maps to its regulation of mTECs. In addition, the effect of LTβR on mTECs is separable from its importance in thymic tolerance, further suggesting that LTβR mediates tolerance by another mechanism. Our observation that LTβR controls thymic cDC1/pDC availability in *Ltbr^−/−^* mice provides an explanation for this, and fits well with the autoimmune phenotype of these mice and the need for both intrathymic (cDC1) and extrathymic (pDC) cells in tolerance induction (Proietto et al., 2008; Hadeiba et al., 2012; Perry et al., 2014). Interestingly, although thymic DCs regulate both negative selection and T-reg development, DC defects in *Ltbr^−/−^* mice correlate with a selective reduction in thymocytes undergoing negative selection. This emphasizes the importance of DC-mediated negative selection as a mechanism of thymic tolerance, which is in agreement with a quantitative requirement for DCs in thymocyte deletion (Anderson et al., 1998; Kroger et al., 2016). Moreover, although mTECs influence thymic DCs (Lei et al., 2011; Spidale et al., 2014), the role of LTβR in formation of the thymic DC pool maps to non-TEC stroma. Indeed, similar to *Ltbr^−/−^* mice, we saw thymic DC defects in LTβR^MES^ mice in which LTβR was deleted in the mesenchyme, demonstrating a role for these cells in the regulation of intrathymic DCs. How mesenchymal cells control thymic DCs is not currently known, although it is interesting that in both mesenchyme and endothelium LTβR regulates expression of chemokines and adhesion molecules (Lkhagvasuren et al., 2013; Lucas et al., 2016) that may aid thymus entry of DCs/DC progenitors.

In summary, we examined properties of the thymus medulla that enable it to act as a highly effective and essential site for T cell tolerance. Our finding that LTβR controls thymic DCs identifies a new role for this TNFRSF member in regulating thymus function and demonstrates the importance of negative selection during tolerance induction. Moreover, that correct medulla formation can be separated from its ability to support thymic tolerance raises the possibility that typical medullary topology is closely associated with other functions of this site. These may include aspects of postselection αβT cell development (Webb et al., 2016; Xing et al., 2016) and the regulation of thymic emigration (Zamora-Pineda et al., 2016), as well the medulla’s role in supporting nonconventional T cell lineages (Roberts et al., 2012; White et al., 2014; Jenkinson et al., 2015).

## Materials and methods

### Mice

All mice were age 8–12 wk on a C57BL/6 background: WT (CD45.2), WT BoyJ (CD45.1), germline LTβR-deficient (*Ltbr*^−/−^; Fütterer et al., 1998), *Foxn1^Cre^* (Gordon et al., 2007), *Wnt1^Cre2^* (Lewis et al., 2013), and *Ltbr^fl/fl^* (Wang et al., 2010) mice. The latter were crossed with *Foxn1^Cre^* mice to obtain LTβR^TEC^ mice and with *Wnt1^Cre^*^*2*^ mice to generate LTβR^MES^ mice. In all experiments, WT C57/BL6 controls were used for *Ltbr^−/−^* mice, and *Foxn1^Cre^* or *Wnt1^Cre2^* mice were used as controls for LTβR^TEC^ and LTβR^MES^ mice, respectively. Mice were housed at the University of Birmingham Biomedical Services Unit. All experimental procedures were approved by the Birmingham Animal Welfare and Ethical Review Body and were performed in accordance with UK Home Office regulations.

### Antibodies and cell sorting

For stromal analysis, thymus samples were digested in collagenase dispase and DNase I (Sigma-Aldrich). Samples were stained with antibodies to the following (from eBioscience unless stated otherwise): CD45 APC (30-F11), EpCAM-1 PerCp Cy5.5 (G8.8), Ly51 PE (6C3), MHCII IA/IE Pacific Blue (M5/114.15.2), Aire Alexa Fluor 488 (5H12), anti-Fezf2 (F441; IBL), and CD80 BV605 (16-10A1; BioLegend). Rabbit anti-CCL21 (Lifespan Biosciences) was detected using Alexa Fluor 647–conjugated goat anti-rabbit (Life Technologies); biotinylated anti-LTβR (3C8) and biotinylated UEA-1 (Vector Laboratories) were detected using streptavidin PE Cy7. For thymocyte analysis, thymic tissue was mechanically disrupted and stained with antibodies to CD4 BV711 (RM4-5; BioLegend), CD8 BV510 (53-6.7; BioLegend), TCRβ APC-Cy7 (H57-597), CD25 APC (PC61.5), CCR7 PE (4B12), and CD5 Biotin (53-7.3) and detected with streptavidin PE Cy7, CD69 PerCp Cy5.5 (H1.2F3), CD3ε PE (clone 145-2C11), Foxp3 FITC (FJK-16s), and cleaved Caspase-3 PE (Asp175, 5AIE; Cell Signaling Technology). Intracellular staining was performed using the Foxp3/transcription factor staining buffer set (eBioscience) according to the manufacturer’s instructions. To detect activated αβT cells in salivary glands, submandibular salivary glands were digested with 30 µg/ml Liberase (Roche) and stained with antibodies to CD45, CD4, CD8, TCRβ, CD69, and CD44 (IM7; eBioscience). For DC analysis, samples were digested using collagenase D and DNase I and stained with antibodies to the following: CD45.2 BV785 (104; BioLegend), PDCA-1 Pacific Blue (129C1; BioLegend), CD11c PeCy7 (N418), Sirpα PE (P84), and CD45.1 APCCy7 (A20). A lineage cocktail containing FITC-labeled antibodies to CD3 (145-2C11), CD19 (eBio1D3), and NK1.1 (PK136) was also used.

### Autoantibody detection

Autoantibodies were detected in serum samples obtained from 8–12-wk-old WT, *Ltbr^−/−^*, *Foxn1^Cre^*, and *Ltbr^fl/fl^* mice using a NovaLite rat liver, kidney, and stomach multicomposite kit (Innova Diagnostics). In brief, tissue sections were incubated with 1/80 sera from the indicated mouse strains at room temperature followed by detection with goat (Fab)_2_ anti–mouse IgG(H+L) FITC (SouthernBiotech). Images were acquired with a DM6000 microscope (Leica Microsystems). Quantification of autoantibodies was performed by two independent staff members based on positive staining intensity on an arbitrary scale of 1–6.

### Histology

Liver and salivary gland samples from WT, *Ltbr^−/−^*, *Foxn1^Cre^*, and LTβR^TEC^ mice were embedded in OCT compound (Sakura Finetek), snap frozen, and sectioned to a thickness of 7 µm. Sections were fixed in acetone for 10 min at 4°C and stained with hematoxylin and eosin, and images were acquired with a Axio ScanZ1 microscope (Zeiss). Cellular infiltrates were quantified by counting cell foci on 3–5 sections per tissue 30–40 µm apart, with infiltrates scored as more than 25 cells clustered together. Software used for analysis was Zeiss Zen Blue.

### Confocal microscopy

Snap-frozen thymus tissues were mounted in OCT, sectioned at 7 µm, and fixed in acetone. The following reagents were used: anti-Aire Alexa Fluor 488 (clone 5H12), anti-Fezf2 (F441, IBL), donkey anti–rabbit IgG Alexa Fluor 594 (Thermo Fisher Scientific), ERTR5 (gift from W. van Ewijk, Leiden University Medical Centre, Leiden, Netherlands), goat anti–rat IgM Alexa Fluor 647 (Thermo Fisher Scientific), goat anti–rat IgM Alexa Fluor 488 (Thermo Fisher Scientific), anti-CD11c Biotin (HL3; BD), anti-CD8 Biotin (53-6.7), and streptavidin Alexa Fluor 555 (Thermo Fisher Scientific). All confocal microscopy was performed on a Zeiss Zen 780 microscope. For quantitation, three to four thymus sections were stained, five images were acquired of medullary and cortical areas, and CD11c^+^ cells were enumerated. All imaging analysis was conducted using Zeiss Zen Black software.

### Quantitation of medullary areas

In frozen thymus sections, boundaries of ERTR5^+^ medullary areas were identified using Zeiss Zen Blue software. Three sections per thymus were analyzed, with a minimum of three mice per strain. Medullary regions were categorized according to area in square millimeters, and the mean number of medullas within each size category was calculated.

### BrdU incorporation in thymic DCs

Adult mice were injected i.p. with 1.5 mg BrdU, and tissues were harvested 18 h later. Thymic DC subsets were identified by flow cytometry, and BrdU incorporation was revealed after cell permeabilization using BrdU flow kit (BD PharMingen) and staining with an APC-conjugated anti-BrdU antibody (MoBU-1).

### BM chimeras

BM cells from femurs and tibias of *Ltbr^−/−^* (CD45.2), C57BL/6 (CD45.2), or BoyJ (CD45.1) mice were T cell depleted using PE-labeled anti-CD3 and anti-PE microbeads and LS columns (Miltenyi Biotec). Host mice were lethally irradiated (two split doses of 500 rad) and reconstituted on the second day of irradiation with 5 × 10^6^ T cell–depleted BM cells from donor mice. Mice were analyzed 8 wk after reconstitution.

### Fetal thymus organ cultures

Embryonic day 15 lobes were cultured with 1.35 mM 2 deoxyguanosine (2dGuo) for 7 d (Cowan et al., 2013). 2dGuo FTOCs were stimulated with 2 µg/ml each of anti-RANK (R&D Systems) or anti-LTβR (Banks et al., 2005) for 4 d. Lobes were digested using 0.25% trypsin/0.02% EDTA (Sigma-Aldrich) and depleted of any remaining CD45^+^ with Dynabeads (Anderson et al., 1993). Cells were then snap frozen for quantitative PCR (qPCR) or permeabilized and stained with antibodies to CD45, EpCAM1, Ly51, Aire, and Fezf2.

### qPCR

qPCR was performed exactly as described (Cowan et al., 2013). mRNA levels were normalized to β-actin, fold levels represent replicate reaction mean (±SEM), and data are typical of at least two independently sorted biological samples. Primer sequences were as follows: β-actin QuantiTect Mm *Actb* 1SG Primer Assay (QT00095242; Qiagen); Aire forward, 5′-TGCATAGCATCCTGGACGGCTTCC-3′, and reverse, 5′-CCTGGGCTGGAGACGCTCTTTGAG-3′; Fezf2 forward, 5′-ACCCAGCTTCCTATCCCCAT-3′, and reverse, 5′-GAGCATTGAACACCTTGCCG-3′; Ccl21 forward, 5′-ATCCCGGCAATCCTGTTCTC-3′, and reverse, 5′-GGGGCTTTGTTTCCCTGGG-3′; Fabp9 forward, 5′-GAATGTGAGCCCCGGAAAGTC-3′, and reverse, 5′-GGATCATTGACCCACCTTCAAA-3′; Ttr forward, 5′-CACCAAATCGTACTGGAAGACA-3′, and reverse, 5′-GTCGTTGGCTGTGAAAACCAC-3′; Krt10 forward, 5′-CAGCTGGCCCTGAAACAATC-3′, and reverse, 5′-AGTTGTTGGTACTCGGCGTT-3′; Spt1 forward, 5′-TACTGAAACTTCTGGAACTGCTGAT-3′, and reverse, 5′-TCGACTGAATCAGAGGAATCAACT-3′; Ins2 forward, 5′-CACCAGCCCTAAGTGATCCG-3′, and reverse, 5′-GCCATGTTGAAACAATAACCTTCCT-3′; and S100a8 forward, 5′-AAATCACCATGCCCTCTACAAG-3′, and reverse, 5′-CCCACTTTTATCACCATCGCAA-3′.

### Statistical analysis

All analyses used GraphPad Prism 6.0. To compare expression levels of LTβR in *Foxn1^Cre^*, *Ltbr^−/−^*, and LTβR^TEC^ mice, one-way ANOVA test was used. In all other cases, we used unpaired Student’s *t* test. Only p-values <0.05 were noted as significant. Nonsignificant differences were not specified. In all figures, error bars represent SEM.

### Online supplemental material

Fig. S1 shows histological and flow cytometric analysis of lymphocyte infiltrates and activated αβT cells in submandibular salivary glands from *Ltbr^−/−^*, LTβR^TEC^, and control mice. Fig. S2 shows flow cytometric analysis of BrdU incorporation in thymic DC subsets from *Ltbr^−/−^*, LTβR^TEC^, and control mice. Fig. S3 shows flow cytometric analysis of DC populations in thymus and spleen of WT:*Ltbr^−/−^* and *Ltbr^−/−^*:WT BM chimeric mice, harvested 8 wk after transplant.

## Supplementary Material

Supplemental Materials (PDF)
