# Supplementary material for: Redefining thymus medulla specialization for central tolerance
Source: J Exp Med. 2017 Nov 6;214(11):3183–95. doi: 10.1084/jem.20171000 (PMC5679166; doi:10.1084/jem.20171000)
Supplement: Supplemental Materials (PDF) [file JEM_20171000_sm.pdf]

The Journal of Experimental Medicine

SUPPLEMENTAL MATERIAL

Cosway et al., https://doi.org/10.1084/jem.20171000

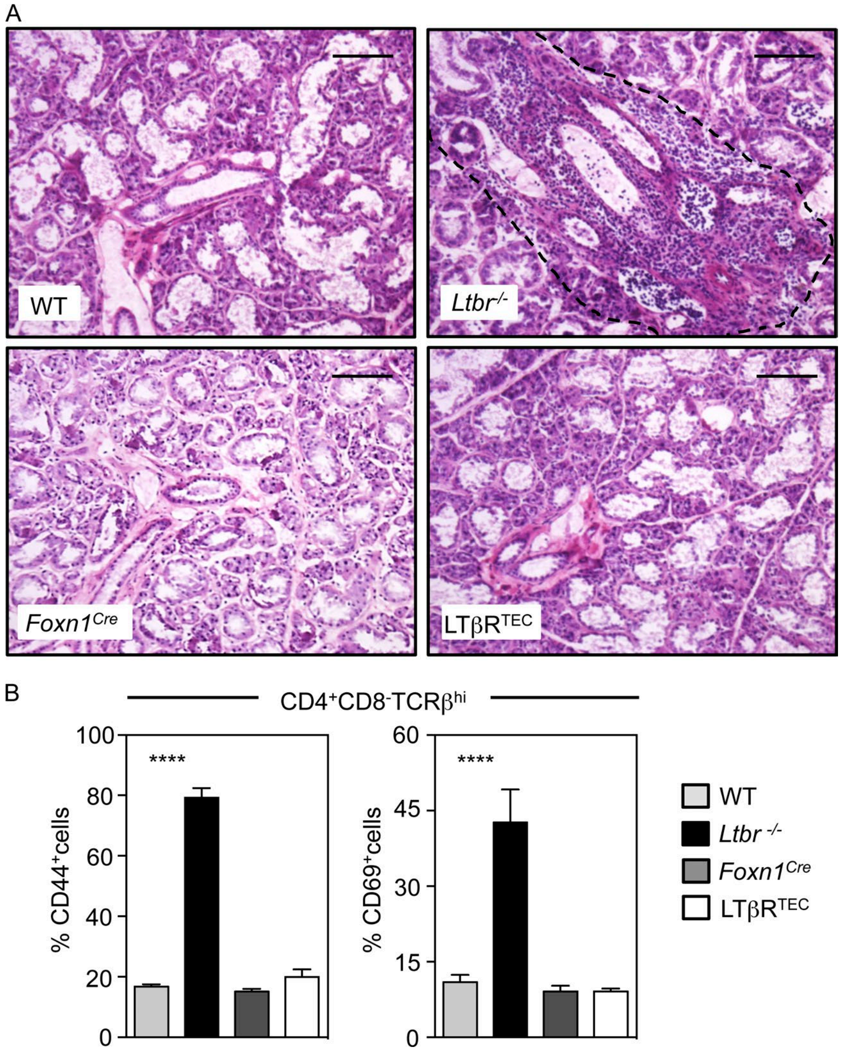

Figure S1. **Symptoms of autoimmunity in the salivary glands of germline *Ltbr*$^{-/-}$ mice but not LTβR^{TEC} mice.** (A) Submandibular salivary gland sections from mice at 8–12 wk of age. Dotted lines indicate lymphocytic infiltrates. Bars, 100 µm. (B) Frequency of CD69^{+} and CD44^{+} activated CD4^{+} αβT cells in digested salivary gland samples from the indicated mouse strains. Data represent two separate experiments, with a minimum of *n* = 6 mice. Error bars indicate SEM. ****, P < 0.0001.

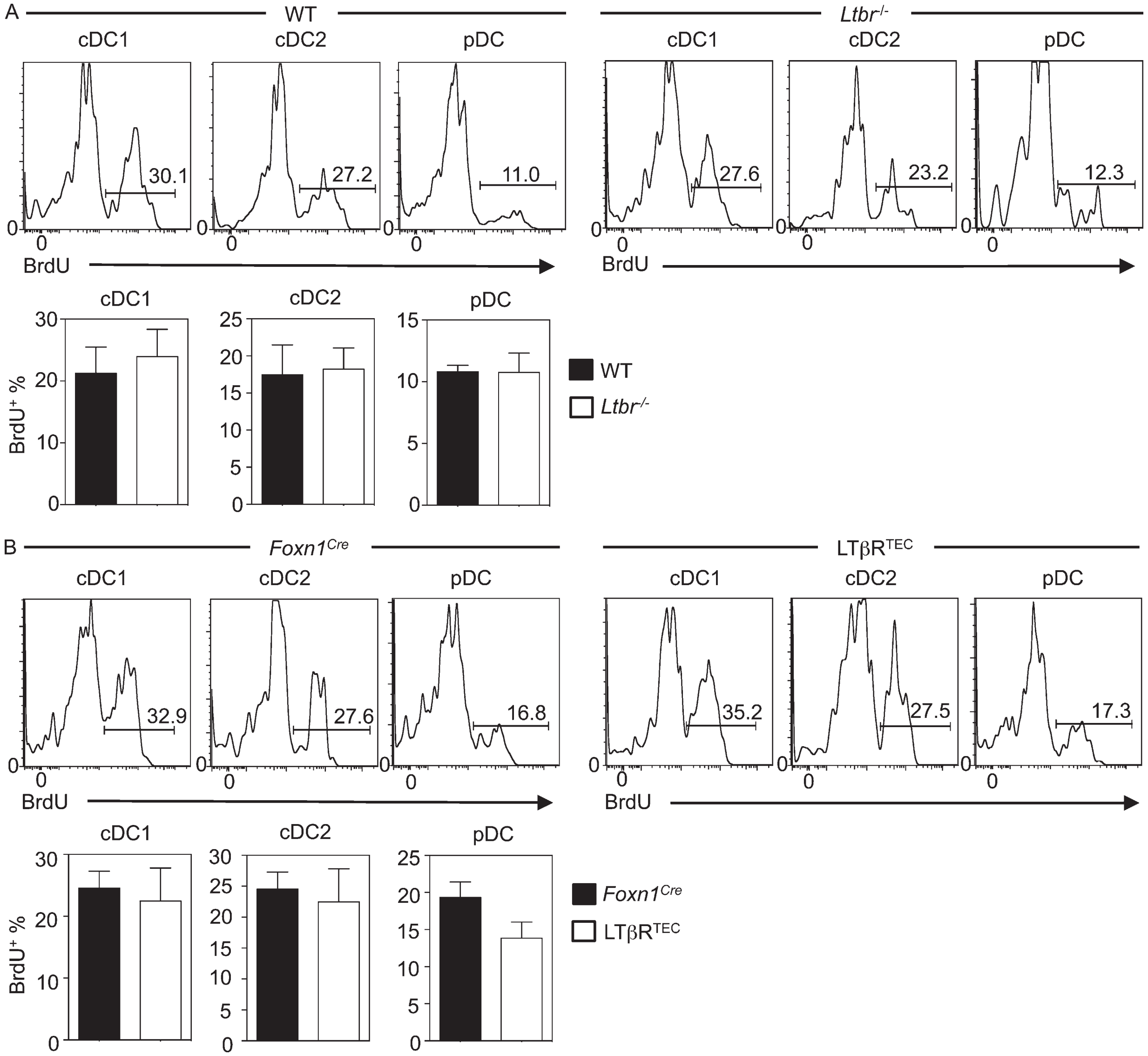

Figure S2. **Analysis of BrdU incorporation in thymic DCs.** BrdU incorporation in cDC1, cDC2, and pDC thymic populations from WT and *Ltbr*$^{-/-}$ mice (A) and LTβR^{TEC} and *Foxn1*^{Cre} mice (B), 18 h after i.p. BrdU injection. Data represent at least three separate experiments, with a minimum of n = 8 for each strain. Error bars represent SEM.

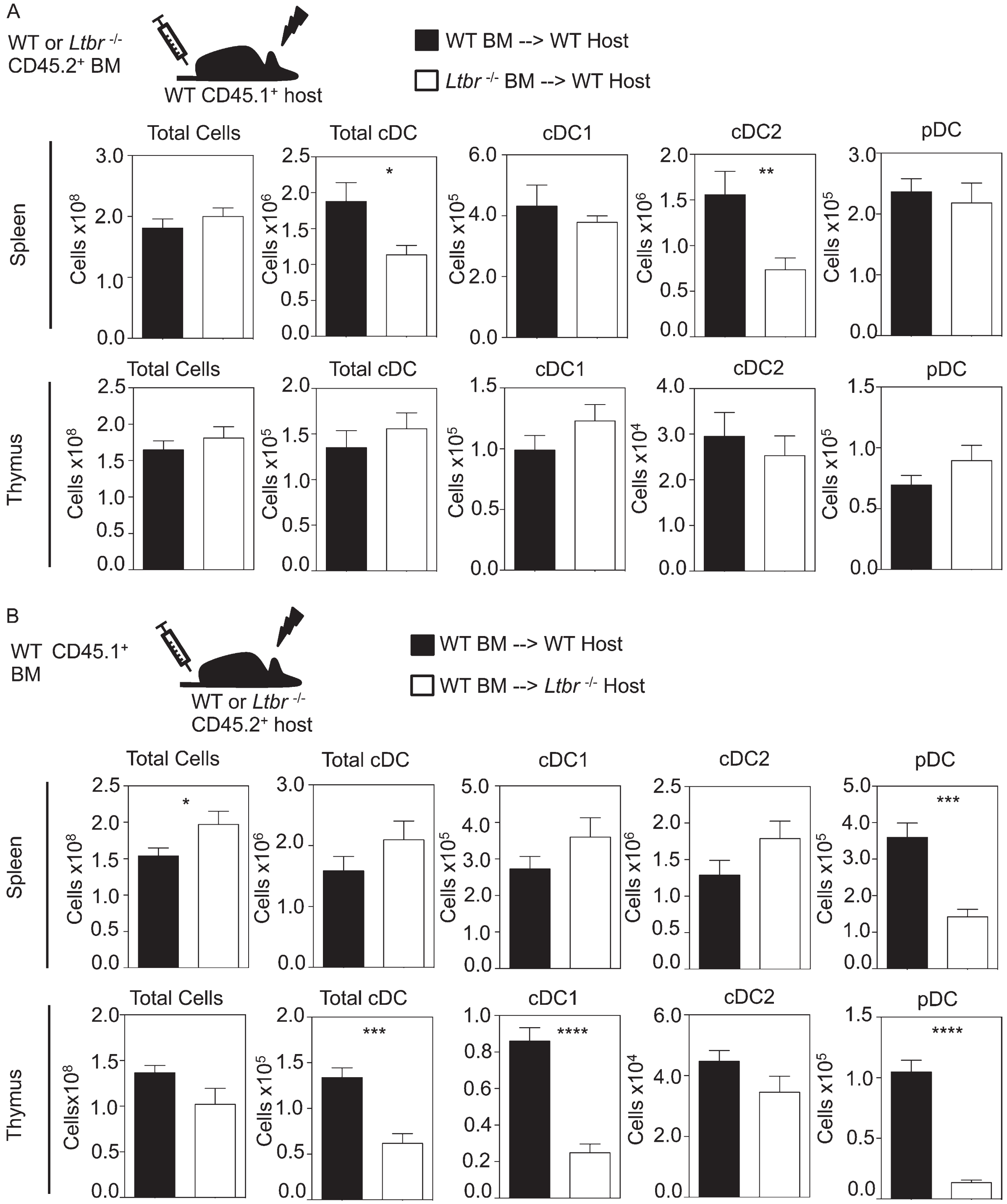

Figure S3. **LTβR expression by thymic stroma controls intrathymic DCs.** (A) Generation of BM chimeras with WT hosts lethally irradiated and reconstituted with WT (black bar) or *Ltbr*$^{-/-}$ (white bar) BM. Quantitation of DCs is shown for spleen and thymus. Data representative of at least $n = 8$. (B) Reverse chimeras were generated with WT or *Ltbr*$^{-/-}$ hosts lethally irradiated and reconstituted with WT BM. DC numbers in spleen and thymus are shown. Analysis was performed 8 wk after reconstitution. Data represent at least three independent experiments; minimum of $n = 10$. Error bars indicate SEM. *, $P < 0.05$; **, $P < 0.01$; ***, $P < 0.001$; ****, $P < 0.0001$.
